# Whole-Mount Adult Ear Skin Imaging Reveals Defective Neuro-Vascular Branching Morphogenesis in Obese and Type 2 Diabetic Mouse Models

**DOI:** 10.1038/s41598-017-18581-7

**Published:** 2018-01-11

**Authors:** Tomoko Yamazaki, Wenling Li, Ling Yang, Ping Li, Haiming Cao, Sei-ichiro Motegi, Mark C. Udey, Elise Bernhard, Takahisa Nakamura, Yoh-suke Mukouyama

**Affiliations:** 1 Laboratory of Stem Cell and Neuro-Vascular Biology, Genetics and Developmental Biology Center, National Heart, Lung, and Blood Institute, National Institutes of Health, 10 Center Drive, Bethesda, MD 20892 USA; 2 Laboratory of Obesity and Metabolic Diseases, Center for Molecular Medicine, National Heart, Lung, and Blood Institute, National Institutes of Health, 10 Center Drive, Bethesda, MD 20892 USA; 3 Dermatology Branch, National Cancer Institute, 10 Center Drive, Bethesda, MD 20892 USA; 4 Divisions of Endocrinology, Cincinnati Children’s Hospital, 3333 Burnet Avenue, Cincinnati, OH 45229 USA; 5 Divisions of Developmental Biology, Cincinnati Children’s Hospital, 3333 Burnet Avenue, Cincinnati, OH 45229 USA; 6 Present Address: Earle A. Chiles Research Institute, Robert W. Franz Cancer Center, Providence Portland Medical Center, Portland, OR 97213 USA; 7 Present Address: Department of Dermatology, Gunma University Graduate School of Medicine, Maebashi, Gunma, 371-8511 Japan; 8 Present Address: Division of Dermatology, Department of Medicine, Washington University School of Medicine, St. Louis, MO 63110 USA

## Abstract

Obesity and type 2 diabetes are frequently associated with peripheral neuropathy. Though there are multiple methods for diagnosis and analysis of morphological changes of peripheral nerves and blood vessels, three-dimensional high-resolution imaging is necessary to appreciate the pathogenesis with an anatomically recognizable branching morphogenesis and patterning. Here we established a novel technique for whole-mount imaging of adult mouse ear skin to visualize branching morphogenesis and patterning of peripheral nerves and blood vessels. Whole-mount immunostaining of adult mouse ear skin showed that peripheral sensory and sympathetic nerves align with large-diameter blood vessels. Diet-induced obesity (DIO) mice exhibit defective vascular smooth muscle cells (VSMCs) coverage, while there is no significant change in the amount of peripheral nerves. The leptin receptor-deficient *db/db* mice, a severe obese and type 2 diabetic mouse model, exhibit defective VSMC coverage and a large increase in the amount of smaller-diameter nerve bundles with myelin sheath and unmyelinated nerve fibers. Interestingly, an increase in the amount of myeloid immune cells was observed in the DIO but not *db/db* mouse skin. These data suggest that our whole-mount imaging method enables us to investigate the neuro-vascular and neuro-immune phenotypes in the animal models of obesity and diabetes.

## Introduction

The prevalence of obesity has increased worldwide in the past 50 years and is closely associated with an increase of insulin-independent type 2 diabetes. Diabetic neuropathy, or damage to the peripheral nervous system, is the most common complication of diabetes, occurring in up to 60% of all diabetic patients^1^. Diabetic neuropathy is characterized by sensory symptoms, such as pain and weakness, with or without numbness. The sensory symptoms are caused by a progressive distal-to-proximal degeneration of peripheral sensory nerves. Despite efforts to make an early diagnosis and halt the progression, these is no effective treatment available beyond pain relief and glucose control^2^.

Though diabetes is the most common recognized cause, human epidemiology and animal studies have demonstrated a link between obesity and neuropathy: diabetic neuropathy in type 2 diabetes appears to associate with prediabetes, obesity, and metabolic syndrome^3^. Indeed, obesity and type 2 diabetes involve chronic inflammation^4,5^. Together with the onset of glucose intolerance, high-fat diet (HFD) induces inflammatory cytokine expression, resulting in the activation of the innate immune system and macrophage infiltration. Whether such a peripheral immune cell activation is a primary cause of nerve injury remains to be elucidated^3^.

Analysis of the anatomical characteristics of the peripheral nervous system and vasculature may provide insights into development of diabetic neuropathy. A three-dimensional high-resolution imaging technique is necessary to appreciate the pathogenesis of peripheral nerves and blood vessels. Using whole-mount imaging of embryonic limb skin^6,7^, we have previously demonstrated the process of sensory nerve-artery alignment^8–10^. We herein introduce a novel technique for whole-mount imaging of adult ear skin to analyze neuro-vascular branching morphogenesis at a cellular level. In the adult ear skin, peripheral sensory and sympathetic nerves align with large-diameter blood vessels. In the peripheral ear skin region, diet-induced obesity (DIO) mice exhibit defective VSMC coverage, while there is no significant change in the amount of nerves. Leptin receptor-deficient (*db/db*) type 2 diabetic mice exhibit defective VSMC coverage and a significant increase in the amount of smaller-diameter myelinated nerve bundles and unmyelinated nerve fibers in the periphery. Interestingly, an increase in the amount of myeloid immune cells was observed in the DIO, but not in *db/db* skin, suggesting that leptin signaling regulates the infiltration of these cells. These data suggest that our whole-mount imaging method enables us to investigate the neuro-vascular and neuro-immune phenotypes in mouse models of obesity and diabetes.

## Results

### Visualization of adult ear skin innervation and vascularization

**Figure 1:**
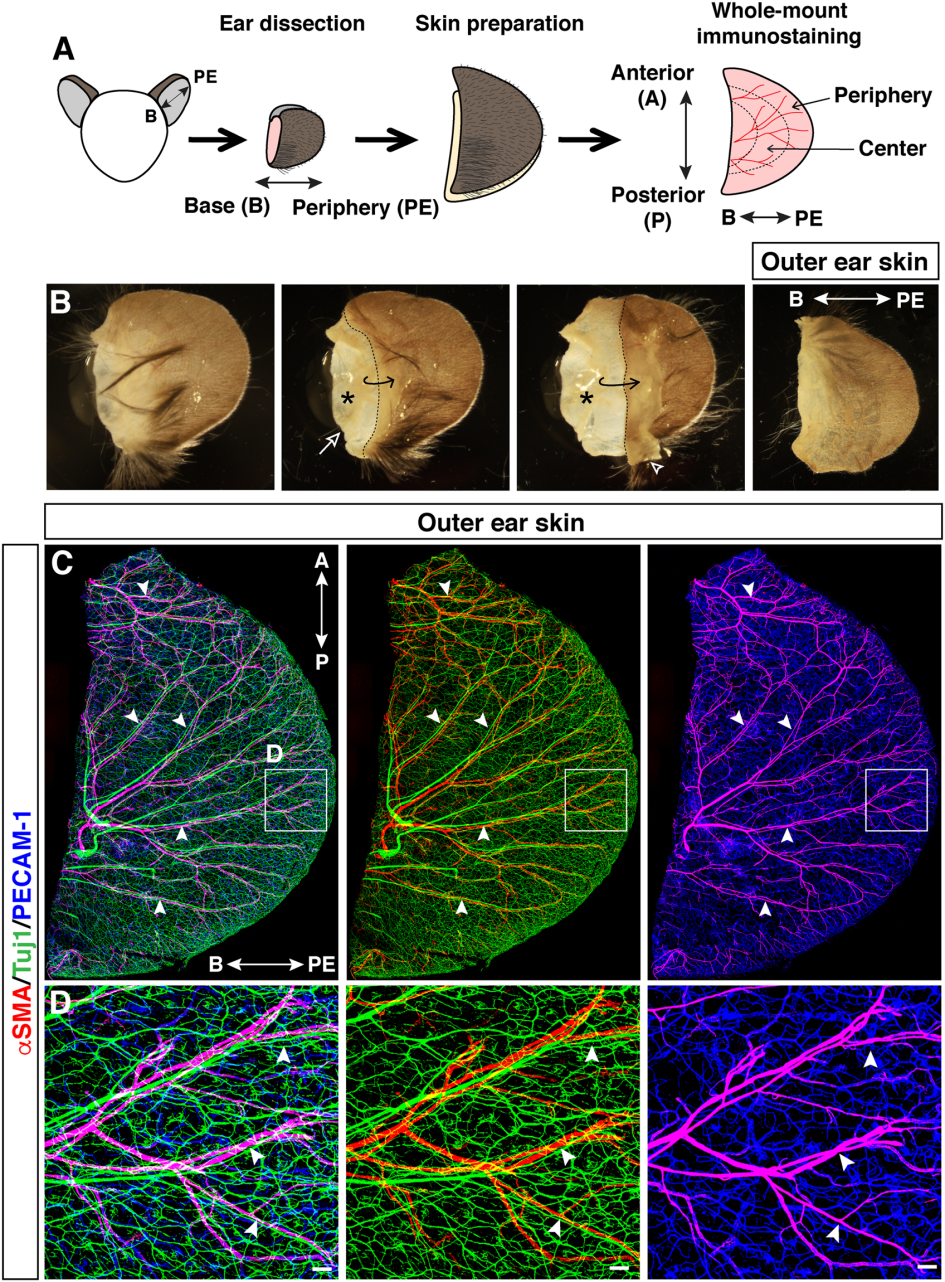
Whole-mount immunofluorescence confocal microscopy of adult outer ear skin. (**A**) Diagram of experimental procedure for adult ear skin whole-mount staining. The orientation of base (B)-periphery (PE) axis and anterior (A)-posterior (P) axis is shown. (**B**) Procedure of the ear skin preparation. Outer ear skin (open arrowhead) and inner ear skin (open arrow) were peeled away from the intervening cartilage (asterisk). Hairs and connective tissues were removed before staining. (**C**,**D**) Outer ear skin was stained with antibodies to a vascular smooth muscle cell (VSMC) marker αSMA (red), a pan-neuronal marker Tuj1 (green), and a pan-endothelial marker PECAM-1 (blue). The boxed regions of (**C**) in the peripheral region of the outer ear skin are magnified in (**D**). Arrowheads indicate the alignment of peripheral nerves and VSMC-covered large-diameter blood vessels. Scale bars, 100 µm.

To study the intricate vascular and nerve branching morphogenesis and patterning in adult skin, we focused on the ear skin as a model because of the simple and reproducible dissection for whole-mount immunohistochemical analysis. The outer and inner parts of the ear skin were peeled away from the intervening cartilage (Fig. 1A,B, Supplemental Fig. 1A,B). Our high-resolution whole-mount immunohistochemistry of the ear skin was performed with antibodies to platelet endothelial cell adhesion molecule (PECAM-1) as a pan-endothelial cell marker, neuron-specific class III β-tubulin (Tuj1) as a pan-neuronal marker, and α smooth muscle actin (αSMA) as a VSMC marker. This method enabled us to visualize peripheral nerves and blood vessels in the deeper dermis (reticular dermis) using confocal microscopy. The outer skin has large-diameter nerve bundles (20–50 µm) aligned with remodeled large-diameter blood vessels covered with αSMA^+^ VSMCs (20–60 µm) (Fig. 1C,D). In contrast, the inner skin has smaller-diameter nerve bundles (<20 µm) aligned with smaller-diameter but remodeled blood vessels covered with αSMA^+^ VSMCs (<20 µm) (Supplemental Fig. 1C,D). These data suggest that the congruence of peripheral nerve and blood vessel branching pattern established during embryogenesis is maintained until adulthood. We decided to focus our further analysis on the outer ear skin because of the ease of dissection and clear nerve-vessel alignment throughout the skin.

### Characterization of neuro-vascular wiring in the adult ear skin

**Figure 2:**
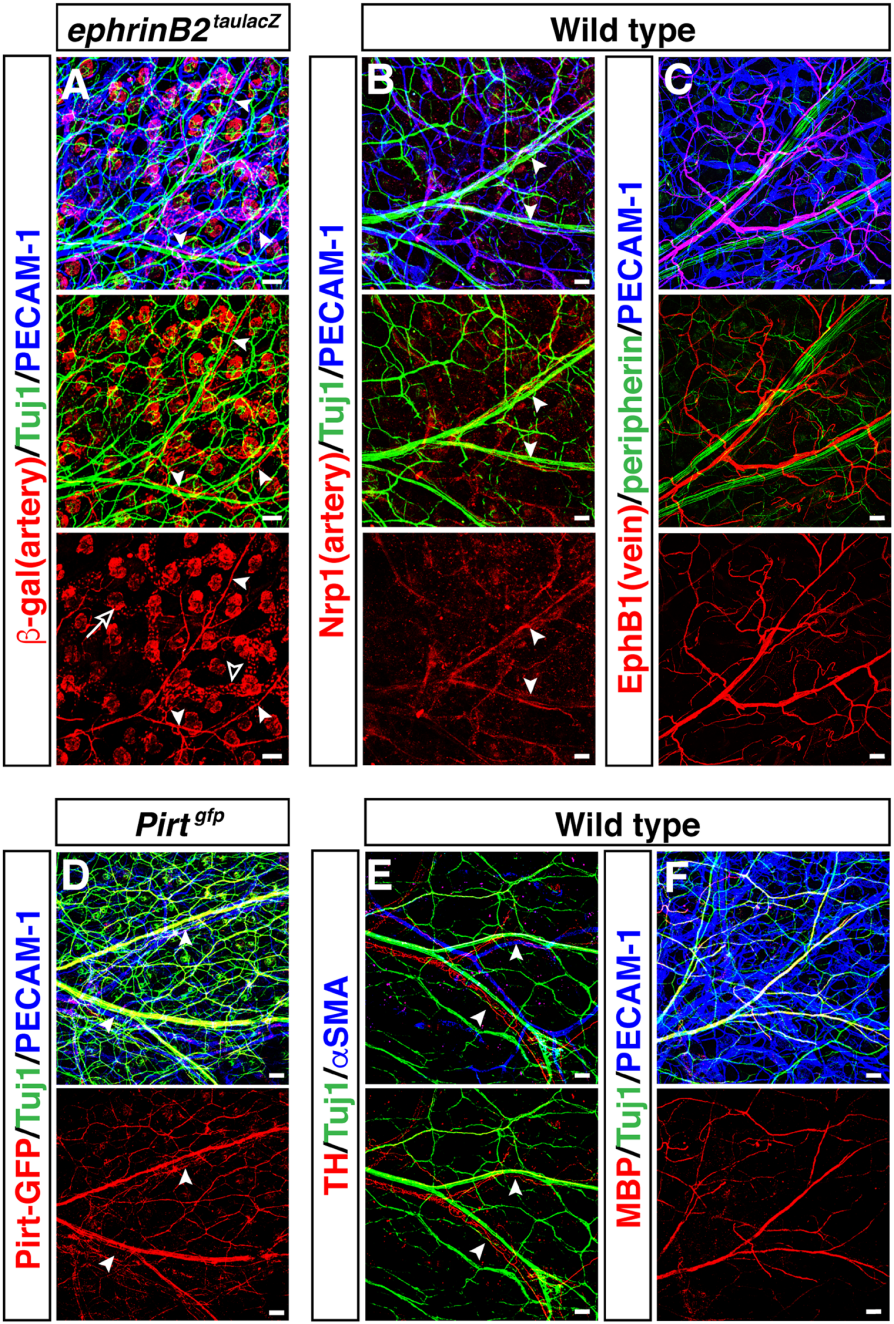
Characterization of neuro-vascular wiring in the adult outer ear skin. (**A**) Whole-mount immunohistochemical analysis of the outer ear skin from adult *ephrinB2* ^*taulacZ*^ mice was performed with antibodies to β-galactosidase (β-gal, red), Tuj1 (green), and PECAM-1 (blue). *ephrinB2* ^*taulacZ*^ expressing arteries align with Tuj1^+^ peripheral nerves (arrowheads). Note that the expression of *ephrinB2* ^*taulacZ*^ was also detected in hair follicles (open arrow) and some lymphatic vessels (open arrowhead). (**B**) Triple labeling with an arterial marker neuropilin1 (Nrp1, red) together with Tuj1 (green) and PECAM-1 (blue) shows that Nrp1^+^ arteries align with Tuj1^+^ peripheral nerves (arrowheads). (**C**) Triple labeling with a venous endothelial cell marker EphB1 (red) together with Tuj1 (green) and PECAM-1 (blue) shows that EphB1^+^ veins align with Tuj1^+^ peripheral nerves in the peripheral region of the outer ear skin. (**D**) Triple labeling for a sensory neuron marker Pirt-GFP (red), Tuj1 (green), and PECAM-1 (blue) in the outer ear skin of adult *Pirt* ^*gfp*^ mice. *Pirt* ^*gfp*+^ sensory nerves align with remodeled blood vessels (arrowheads). (**E**) Triple labeling for a sympathetic neuron marker tyrosine hydroxylase (TH, red), Tuj1 (green), and PECAM-1 (blue). TH^+^ sympathetic nerves associate with VSMC-covered blood vessels (arrowheads). (**F**) Triple labeling for a myelin membrane marker MBP (red), Tuj1 (green), and PECAM-1 (blue). MBP^+^ myelin sheath was detected in peripheral nerves. Representative images from the peripheral region of the outer ear skin are shown. Scale bars, 100 µm.

During angiogenesis, a primary capillary network undergoes intensive vascular remodeling and develops into a hierarchical vascular branching network. Between embryonic day (E) 14.5–15.5, arteries branch alongside peripheral sensory nerves^8,9^, and veins then form adjacent to the arteries^11,12^. We therefore examined the relationship of arteries and veins with peripheral nerves in the adult ear skin. We used *ephrinB2* ^*taulacZ*^ in a *taulacZ* reporter strain and neuropilin1 (Nrp1) as arterial markers^9,13^, in combination with a pan-neuronal marker Tuj1 (Fig. 2A,B). EphB1 was used as a venous marker together with another pan-neuronal marker peripherin^11^ (Fig. 2C). In the adult ear skin, both arteries and veins associated with peripheral nerve bundles throughout the central (data not shown) and peripheral region (Fig. 2A–C). It should be noted that the expression of *ephrinB2* ^*taulacZ*^ was also detected in hair follicles and some lymphatic vessels (Fig. 2A) as previously reported^14,15^.

We next examined the expression of a sensory nerve marker Pirt, phosphoinositide-interacting regulator of transient receptor potential channels^16^, and a sympathetic marker TH, tyrosine hydroxylase^17^, in the adult ear skin. Previous studies demonstrated that from E13.5–15.5, only sensory nerves are present in the limb skin. From E15.5 onwards, sympathetic nerves invade the skin and extend their axons along VSMC-covered large-diameter blood vessels^9,18^. In the adult ear skin, *Pirt* ^*gfp*^-expressing sensory nerve bundles aligned with remodeled blood vessels (Fig. 2D). TH^+^ sympathetic nerve bundles were present in close proximity to VSMC-covered remodeled blood vessels (Fig. 2E). We also detected the expression of myelin basic protein (MBP), a major constituent of the myelin sheath of Schwann cells in peripheral nerves, in medium-to-large-diameter nerve bundles (10–20 µm) as well as some smaller-diameter nerves (<10 µm) in the periphery (Fig. 2F). Combined, these data suggest that both sensory and sympathetic nerves associate with arteries and veins in the adult ear skin.

### Obesity and pre-diabetic type 2 diabetes lead to defective VSMC coverage in the adult ear skin vasculature

**Figure 3:**
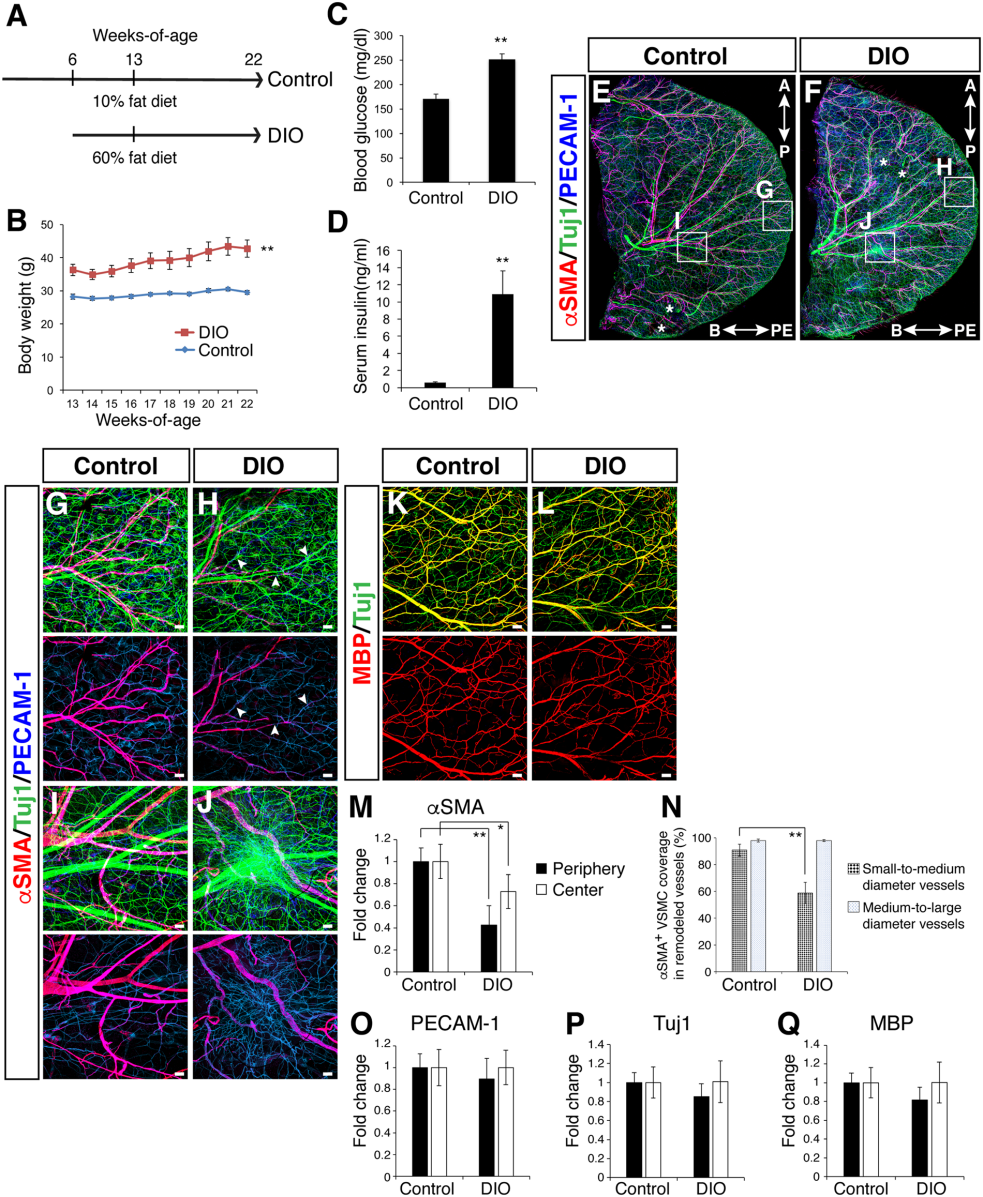
Vascular and neuronal abnormalities in the outer ear skin of adult DIO mice. (**A**) Experimental outline to generate the DIO mice. 6-week-old C57BL/6 J males were fed a HFD (60 Kcal % fat) or normal control diet (10 Kcal % fat) for 7 weeks (n = 5 per group). At 13 weeks-of-age, the mice were transferred from Jackson Laboratory and housed in the NIH animal facility with the same diet until 22 weeks-of-age. (**B**) The effect of HFD or normal control diet on body weight. (**C**) Blood glucose level was elevated in the DIO mice after fasting at 22 weeks-of-age. (**D**) Serum insulin level was elevated in the DIO mice after fasting at 22 weeks-of-age. (**E**,**F**) Whole-mount immunohistochemical analysis of the outer ear skin from control (**E**) and DIO (**F**) mice was performed with antibodies to αSMA (red), Tuj1 (green), and PECAM-1 (blue). Asterisks indicate skin damage from the dissection and/or staining procedure. The boxed regions of (**E**,**F**), peripheral or central region of the outer ear skin, are magnified in (**G**–**J**). The orientation of base (B)-periphery (PE) axis and anterior (A)-posterior (P) axis is shown. (**G**,**H**) Peripheral region of the outer ear skin from control (**G**) and DIO (**H**) mice. αSMA^+^ VSMC (red) coverage was significantly reduced in the periphery of the DIO ear skin. Arrowheads indicate blood vessels (PECAM-1, blue) which lack VSMC coverage (red) but still align with Tuj1^+^ nerves (green). (**I**,**J**) Aberrant bundle of nerves were observed in the central region of the DIO ear skin (**J**), relative to the nerve branching in control ear skin (**I**). (**K**,**L**) Double labeling with MBP (red) and Tuj1 (green) in ear skin from control (**K**) and DIO (**L**) mice. No significant difference in Tuj1^+^ nerves (green) and MBP^+^ myelin sheath (red) between the DIO and control ear skin was observed. (**M**–**Q**) Quantification of αSMA^+^ VSMC coverage (**M**,**N**), PECAM-1^+^ vascular density (**O**), and the amount of Tuj1^+^ nerves (**P**) and MBP^+^ myelin sheath (**Q**). Three regions in the peripheral and central region of each outer ear skin were analyzed and the result was shown as a fold change (**M**,**O**–**Q**). The degree of αSMA^+^ VSMC coverage as a percentage of the total length of small-to-medium and medium-to-large diameter remodeled blood vessels was analyzed (**N**). Data are presented as mean ± S.E.M. Astersisks indicate statistical significance (*P < 0.05; **p < 0.01) according to a two-tailed Student’s *t* test. Scale bars, 100 µm.

We next examined the neuro-vascular wiring in pathological situations including obesity-related nerve disorders. To develop DIO mice, we followed a standard protocol in which C57BL/6 J males were fed a HFD from 6 to 22 weeks-of-age (Fig. 3A). The protocol resulted in a significant increase in body weight (Fig. 3B, 32.2 ± 0.5 g versus 42.2 ± 1.9 g, p = 0.0004), blood glucose (Fig. 3C, 170.6 ± 9.4 mg/dl versus 251.0 ± 12.1 mg/dl, p = 0.0018), and serum insulin level (Fig. 3D, 0.6 ± 0.1 ng/ml versus 10.9 ± 3.1 ng/ml, p = 0.0022).

We then compared branching morphogenesis and patterning of peripheral nerves and blood vessels between DIO and control mice. We carried out whole-mount immunohistochemical analysis of ear skin not only to visualize neuro-vascular branching morphogenesis but also to evaluate vascular density, VSMC coverage, and the amounts of axons and myelin sheath. We found no gross abnormalities in the branching patterns of peripheral nerves and large-diameter blood vessels in the outer ear skin of DIO mice: peripheral nerves aligned with VSMC-covered large-diameter blood vessels (Fig. 3E versus 3F). In the peripheral and central region of the outer ear skin, however, the amount of VSMC coverage in the DIO ear skin vasculature was significantly reduced by up to about 40% of the control (Fig. 3G versus 3H; 3M, p = 0.0005 in the periphery; p = 0.04 in the center), with no significant change in the vascular density (3O, p = 0.2114 in the periphery; p = 0.9954 in the center). We further examined the VSMC coverage in different sizes of remodeled blood vessels: we quantified the degree of VSMC coverage as a percentage of the total length of small-to-medium and medium-to-large diameter blood vessels. Interestingly, we have found a significant reduction in the degree of VSMC coverage in the small-to-medium blood vessels in the DIO, while there was no significant difference in the medium-to-large diameter blood vessels between control and DIO (Fig. 3N, p = 0.000000001 in small-to-medium diameter blood vessels; p = 0.9263 in medium-to-large diameter blood vessels). In contrast, we did not find any significant difference in the total amount of Tuj1^+^ peripheral nerves and MBP^+^ myelin sheath between the DIO and control ear skin (Fig. 3G versus 3H, 3K versus 3L; 3P, p = 0.1496 in the periphery; p = 0.9553 in the center, and 3Q, p = 0.1076 in the periphery; p = 0.9863 in the center). We should note that some large-diameter nerve bundles were collapsed and the axons appeared more de-fasciculated in the central region of the DIO outer ear skin (Fig. 3E versus 3F, 3I versus 3J), though such phenotypes were not consistent across all DIO mice (2 out of 5 DIO mice; no neuronal abnormality was observed in 5 control mice). Taken together, vascular branching morphogenesis, particularly of VSMC coverage, is impaired in the DIO ear skin vasculature, while progressive abnormalities in peripheral nerves might not be detectable consistently in this mouse model of obesity and pre-diabetic type 2 diabetes.

### Increased inflammatory cells in the DIO skin

**Figure 4:**
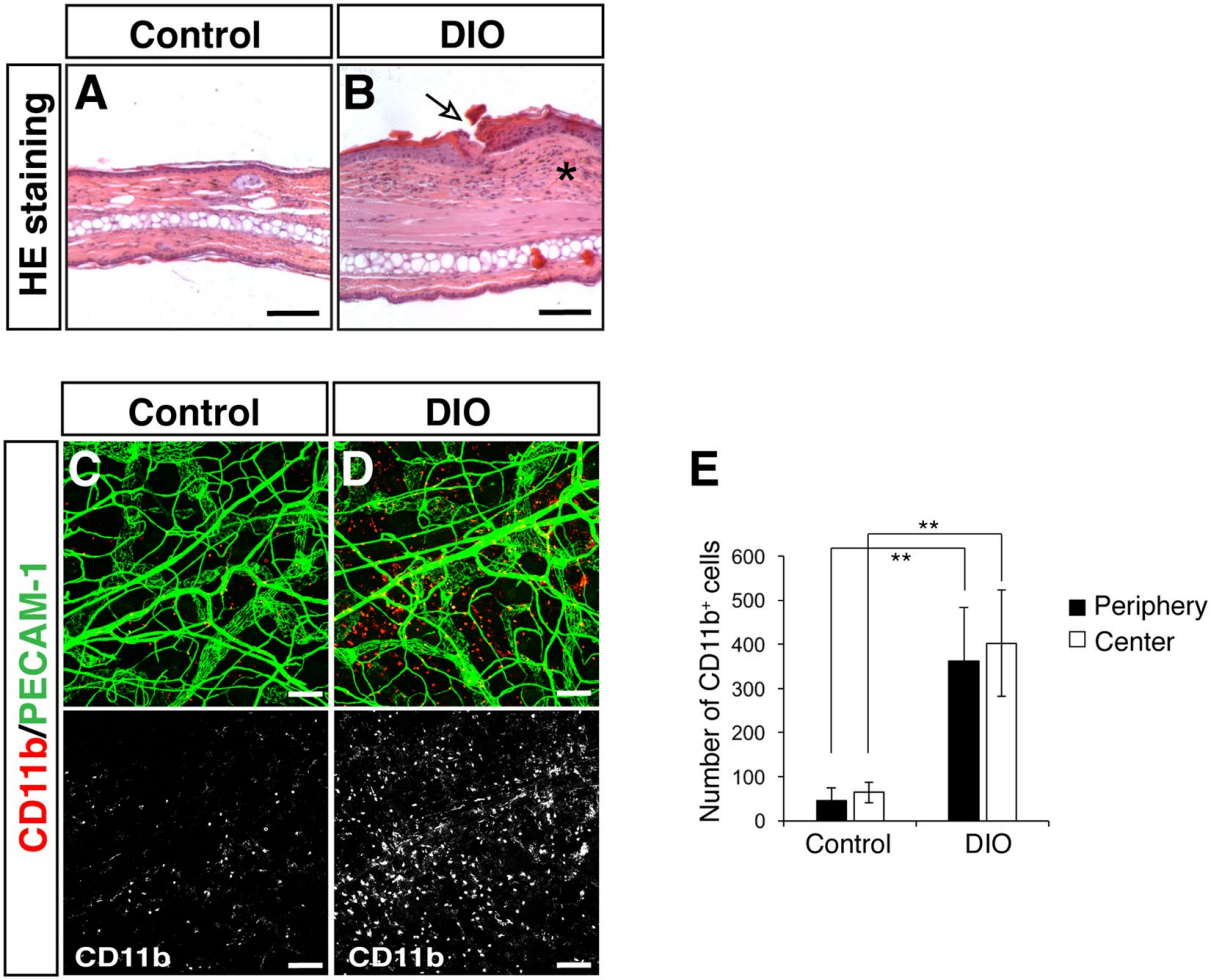
Increased inflammatory cells in the DIO skin. (**A**,**B**) Representative images of hematoxylin-eosin (HE) staining of ear skin from control (**A**) and DIO mice (**B**). Compared to control, DIO ear skin lesions had thickening of the epidermal layer with damage to the epithelial cells (open arrow). The dermis also showed thickening with inflammation (asterisk) and fibrosis beneath the damaged epidermal cells. (**C**,**D**) Double labeling for CD11b (red and white), an inflammatory myeloid cell marker, and PECAM-1 (green). A significant increase in CD11b^+^ inflammatory cells was observed in the periphery of the DIO ear skin (**C** versus **D**, white). (**E**) Quantification of the number of CD11b^+^ inflammatory cells. CD11b^+^ inflammatory cells were manually counted in the peripheral and central region of each outer ear skin. Data are presented as mean ± S.E.M. Astersisks indicate statistical significance (**p < 0.01) according to a two-tailed Student’s *t* test. Scale bars, 100 µm.

Inflammation in obesity is likely to be accompanied by immune cell infiltration. For example, in the DIO mice, previous studies have demonstrated that CD11b^+^/F4/80^+^ macrophages are recruited into the white adipose tissue^19–22^. We thus examined whether macrophages infiltrate into the DIO ear skin. Indeed, the hematoxylin and eosin staining demonstrated that DIO skin had thickening of epidermal layer with some skin lesions. Moreover, thickening of connective tissues was observed in the dermis of DIO skin (Fig. 4A versus 4B). These phenotypes indicate that DIO skin appeared to have inflammation and fibrosis. Consistent with these observaitons, a significant increase in the number of CD11b^+^ inflammatory cells, including macrophages, was observed in the DIO ear skin (Fig. 4C versus 4D; 4E, p = 0.00044 in the periphery; p = 0.00021 in the center). These data suggest that our whole-mount imaging of the reticular dermis in the outer ear skin allows us to investigate the amount and distribution of inflammatory cells in the DIO mice.

### A mouse model of type 2 diabetes reveals impaired VSMC coverage and peripheral nerve projection

**Figure 5:**
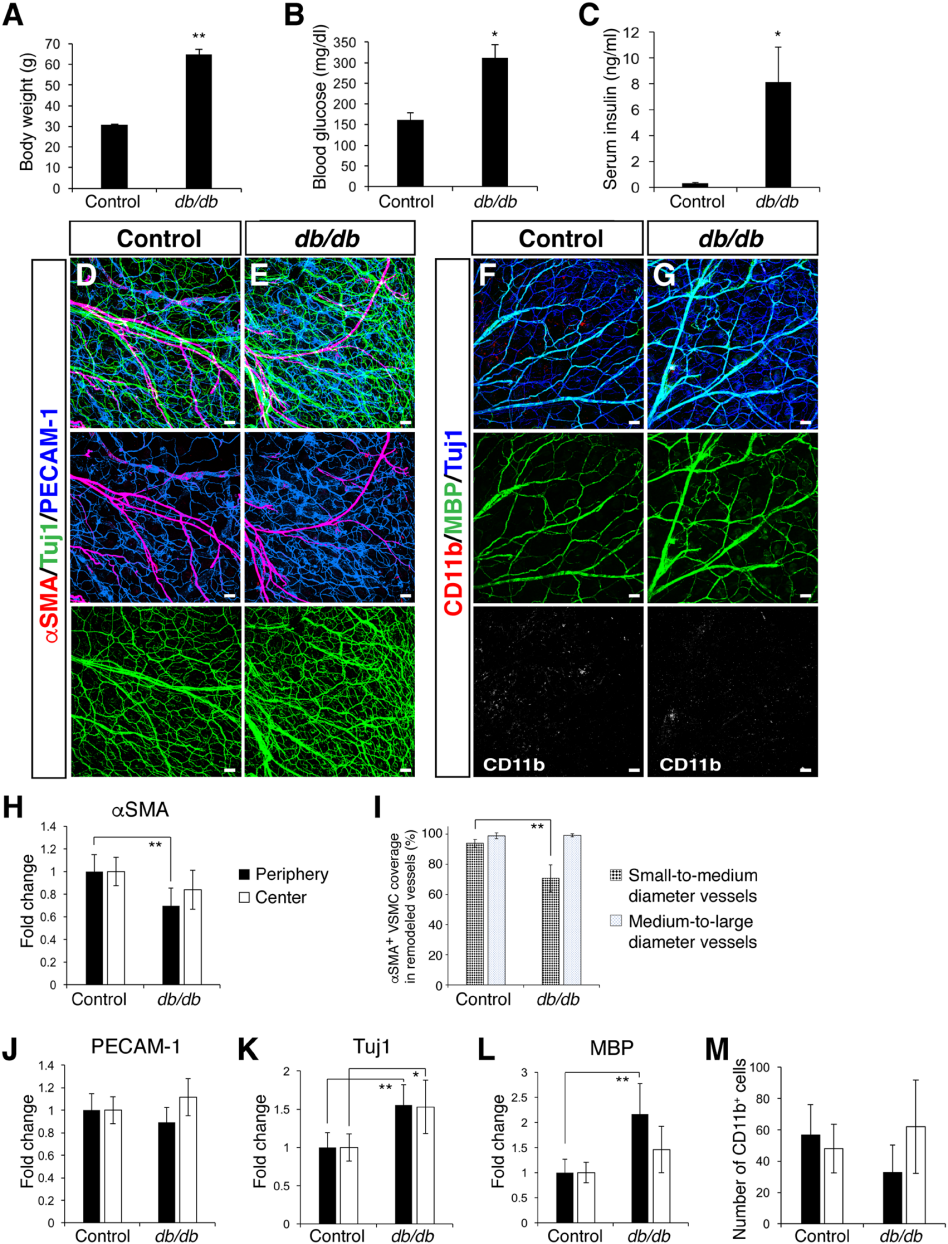
Vascular and neuronal abnormalities in the outer ear skin of adult *db/db* mice. (**A**) Male control and *db/db* mice (n = 3–4 per group) were analyzed at 36 weeks-of-age. A significant increase in body weight was found in *db/db* mice. (**B**) Blood glucose level was elevated in the *db/db* mice after fasting at 36 weeks-of-age. (**C**) Serum insulin level was elevated in the *db/db* mice after fasting at 36 weeks-of-age. (**D**,**E**) Triple labeling with αSMA (red) together with Tuj1 (green) and PECAM-1 (blue) shows a reduction in VSMC coverage (red) and an increase in Tuj1^+^ nerves (green) in the *db/db* mutants. (**F**,**G**) Triple labeling with CD11b (red) together with MBP (green) and Tuj1 (blue) shows an increase in MBP^+^ myelin sheath and Tuj1^+^ nerves (blue) in the *db/db* mutants, while there was no increase in CD11b^+^ inflammatory cells (red). (**H**–**M**) Quantification measurements of αSMA^+^ VSMC coverage (**H**,**I**), PECAM-1^+^ vascular density (**J**), and the amount of Tuj1^+^ nerves (**K**), MBP^+^ myelin sheath (**L**) and the number of CD11b^+^ inflammatory cells (**M**). At least three regions in the peripheral and central region of each outer ear skin were analyzed and the result was shown as a fold change (**H**,**J**–**L**). The degree of αSMA^+^ VSMC coverage as a percentage of the total length of small-to-medium and medium-to-large diameter remodeled blood vessels was analyzed (**I**). Data are presented as mean ± S.E.M. Astersisks indicate statistical significance (*P < 0.05; **p < 0.01) according to a two-tailed Student’s *t* test. Scale bars, 100 µm.

Given that our whole-mount imaging successfully detects impairments of vascular branching morphogenesis in the DIO ear skin, we then asked if these abnormalities are features in another mouse model of obesity and diabetes. We examined branching morphogenesis and patterning of peripheral nerves and blood vessels in leptin receptor-deficient mice (C57BL/6 *db/db*). Leptin receptor deficiency evokes hyperphagia, leading to severe obesity, glucose intolerance, and hyperglycemia^23–25^. Therefore, *db/db* mice are considered a mouse model for obesity-induced type 2 diabetes^23–25^. Indeed, *db/db* mice showed an extensive increase in body weight (Fig. 5A, 30.8 ± 0.4 g versus 64.8 ± 2.5 g, p = 0.0046), blood glucose (Fig. 5B, 161.8 ± 17.5 mg/dl versus 311.3 ± 33.1 mg/dl, p = 0.0263) and serum insulin level (Fig. 5C, 0.3 ± 0.1 ng/ml versus 8.1 ± 2.7 ng/ml, p = 0.018). According to the previous observations that non-fasting blood glucose concentration for diabetes is over 250 mg/dl^26^, the mice were considered to have type 2 diabetes.

Interestingly, we observed a significant increase in the total amounts of smaller-diameter Tuj1^+^/MBP^+^ myelinated nerve bundles and Tuj1^+^/MBP^*−*^ unmyelinated nerve fibers in the *db/db* ear skin (Fig. 5D versus 5E, 5F versus 5G; 5K, p = 0.0004 in the periphery; p = 0.0182 in the center, and 5L, p = 0.0001 in the periphery; p = 0.0979 in the center), though there were no obvious collapsed nerve bundles. We also found defective VSMC coverage in the *db/db* ear skin vasculature (Fig. 5D versus 5E; 5H, p = 0.0035 in the periphery; p = 0.0854 in the center), without any significant change in the vascular density (Fig. 5J, p = 0.2412 in the periphery; p = 0.16 in the center). Likewise, we found a significant reduction in the degree of VSMC coverage in the small-to-medium blood vessels in the *db/db* ear skin vasculature, while there was no significant difference of VSMC coverage in the medium-to-large diameter blood vessels between control and *db/db* (Fig. 5I, p = 0.0000004 in small-to-medium diameter blood vessels; p = 0.7637 in medium-to-large diameter blood vessels). These data suggest that the type 2 diabetic mouse model exhibits not only impaired VSMC coverage but also progressive abnormalities in peripheral nerves.

One intriguing question is whether chronic inflammation might influence branching morphogenesis of peripheral nerves and blood vessels in the adult ear skin. Interestingly, leptin influences inflammation and immunity^27,28^, in addition to its major role as a pleiotropic hormone which regulates appetite, metabolic and endocrine functions. Indeed, no obvious change in the number of CD11b^+^ inflammatory cells was observed in the *db/db* ear skin (Fig. 5F versus 5G, 5M, p = 0.054 in the periphery; p = 0.4407 in the center). Combined, these data suggest that the infiltration of CD11b^+^ inflammatory cells may not be a primary factor to cause neuronal and vascular abnormalities in obesity and type 2 diabetes.

## Discussion

Here we describe a high-resolution whole-mount imaging method in the entire adult mouse ear skin. This method enabled us to visualize branching morphogenesis and patterning of peripheral nerves and blood vessels in adult ear skin, with comprehensive quantification measurements. Ear skin is readily accessible for dissection and subsequent whole-mount imaging of the skin over its entire depth. Thus, it is a straightforward and highly reproducible method that can be applied to compare three-dimensional architecture of peripheral nervous and vascular systems in the skin, in contrast with traditional tissue sectioning methods.

Using our whole-mount imaging method in mouse models of obesity and type 2 diabetes, we discovered a significant reduction of VSMCs in the ear skin vasculature. We also found that the phenotype is more severe in peripheral region than in the central region, which may suggest that smaller-diameter vessels are more affected than large-diameter vessels. Indeed, we found a significant reduction in the degree of VSMC coverage in the small-to-medium blood vessels in the DIO (Fig. 3N) and *db/db* (Fig. 5I) ear skin vasculature, although there was no significant difference of VSMC coverage in the medium-to-large diameter blood vessels between control versus DIO or *db/db*. To our knowledge, this represents the first observation of VSMC reduction in the skin vasculature of hyperglycemic animal models. We associate our findings with the loss of pericytes, another type of mural cell, in diabetic retinopathy^29,30^. Diabetic retinopathy is a common diabetic eye disease that is characterized by increased capillary permiability and occulsion^29,30^. The pericyte loss directly induces inflammatory responses in vascular endothelial cells (ECs) and perivascular infiltration of macrophages^31^. Despite the significance of mural cell deficiency in the progression of diabetic retinopathy, the processes of this deficiency remain elusive. Of note, leptin is known to exert multiple functions including a pro-inflammatory role that activates monocytes and macrophages to produce inflammatory cytokines such as IL-6 and TNF-α^32^. The inflammatory role of leptin can explain our finding that the infiltration of CD11b^+^ immune cells is observed in the DIO but not *db/db* mouse skin. Our studies therefore suggest that defective VSMC coverage occurs independently of inflammation and immune cell infiltration. Further whole-mount analyses of animals harboring genetic modulation associated with VSMC functions (e.g. VSMC coverage and EC-VSMC interations) would reveal the role of VSMCs in the neuro-vascular pathogenesis associated with obesity and type 2 diabetes.

A significant increase in the amount of peripheral nerves, particularly smaller-diameter myelinated nerve bundles and unmyelinated nerve fibers, was observed in the *db/db* mutants. It has been reported that neuropathy of small nerve fibers is an early feature of peripheral nerve injury in diabetes, and larger myelinated fibers are relatively unaffected^33^. In contrast with *db/db* mutants, DIO mice exhibit de-fasciculation of large bundles of peripheral nerves, though the phenotype was observed in only some of the DIO mice. Our observation that some large-diameter nerve bundles were collapsed and the axons appeared more de-fasciculated may mimic a symptom of diabetic neuropathy in patients^2^. Small fiber-predominant neuropathy is an early manifestation and can be difficult to diagnose. Patients often progress from a small fiber-predominant neuropathy to a diabetic neuropathic pain. Combined, our data suggest that abnormal morphogenesis in peripheral nerves is associated with type 2 diabetes, while the progressive abnormalities in the nerves may not be associated with obesity and pre-diabetic type 2 diabetes. One intriguing question is whether there is a link between vascular and neuronal abnormalities. Defective VSMC coverage might lead to defective vessel integrity and capillary leakage, which could damage neighboring peripheral nerves. The mechanisms underlying the influence of hyperglycemia on peripheral nerve abnormalities are an interesting topic for future investigation.

The present studies clearly demonstrate that a novel whole-mount imaging method of adult ear skin facilitates the analysis of branching morphogenesis and patterning of peripheral nerves and blood vessels, as well as immune cell distribution, in pathological conditions such as obesity and diabetes. This approach could expedite the research of such morphological abnormalities and screen drugs to treat them.

## Methods

### Experimental animals and Diet-Induced Obesity

C57BL/6 J mice were purchased from the Jackson Laboratory. C57BL/6 J males were placed a HFD (60% fat, 20% protein, and 20% carbohydrate kcal; Research Diets #D12492) for a diet induced obese model or a normal control diet (10% fat, 20% protein, 70% carbohydrate kcal; Research Diets #D12450B) beginning at 6 weeks-of-age ad libitum with free access to water for 7 weeks at the Jackson Laboratory. The mice were transferred and housed in the National Institutes of Health (NIH) animal facility with the same diet until 22 weeks-of-age. 36 week-old *db/db* homozygous mutants^23–25^ (The Jackson Laboratory) and their control (+/+ or *db/*+) mice were housed in the Nakamura laboratory. *Pirt* ^*gfp*^ mice^16^ and *EphrinB2* ^*taulacZ*^ mice^13^ have been reported elsewhere. All experiments were performed under approval from the National Heart, Lung, and Blood Institute (NHLBI) Animal Care and Use Committee.

### Body weight, blood glucose, and serum insulin measurement

Weights were measured weekly at the same time of the day. Mice were fasted for 4–6 hours before measuring blood glucose level using Breeze2 (bayer). Blood samples were collected after cardiac puncture when mice were euthanized. Serum insulin level was measured by Mouse Ultrasensitive Insulin ELISA according to manufacturer’s protocol (ALPCO). These experiments were performed under approval from the NHLBI Animal Care and Use Committee.

### Immunohistochemistry and imaging

Ear skin from adult mice was dissected, fixed in 4% paraformaldehyde (Electron Microscopy Sciences) in phosphate buffered saline (PBS) for 1 hour at 4 °C, and washed 3 times in PBS. Connective tissues, fat, and hairs were removed before staining. The samples were incubated in 0.2% TritonX-100 and 10% heat inactivated goat serum (Thermo scientific) in PBS, with diluted primary antibodies, overnight at 4 °C. The primary antibodies were: anti-PECAM-1 antibody (clone MEC13.3, BD Pharmingen, 1:300) to detect endothelial cells; β-gal antibody (Cappel, 1:500) to detect *ephrinB2* ^*taulacZ*^ as an arterial endothelial cell marker; Cy3-conjugated anti-αSMA antibody (clone 1A4, Sigma, 1:500) to detect vascular smooth muscle cells; anti-peripherin (Chemicon 1:1000) and anti-Tuj1 antibody (Covance, 1:500; abcam, 1:500) to detect peripheral nerve fibers; anti-MBP (Abcam 1:200) to detect myelin membrane in peripheral nerves; anti-TH (Chemicon 1:100) to detect sympathetic axons; anti-GFP (abcam, 1:500) to detect *Pirt* ^*gfp*^ as a sensory neuron marker; anti-CD11b (AbD Serotec, 1:100) to detect inflammatory cells. After the staining, samples were washed 3 times for 10 min in 2% goat serum with 0.2% TritonX-100 in PBS at room temperature with rotation. For immunofluorescent detection, the samples were incubated in blocking buffer containing either Alexa-488-, Alexa-594-, Cy3- or Alexa-647-conjugated secondary antibodies (Thermo scientific 1:250 or Jackson, 1:300). After 3 washes as described previously, the samples were mounted on slide in mounting media (Prolong Gold, Thermo scientific). The inner surface of the ear skin was in contact with the cover slip. All confocal microscopy was carried out on a Leica TCS SP5 confocal (Leica).

For quantification measurements, at least three regions in the periphery and center of each outer ear skin were analyzed. The threshold level was set to minimize background noise, and then the pixel intensity values for positive-stained area were measured using NIH ImageJ software (NIH). The percentage of positive pixel intensity values in the DIO or *db/db* samples was normalized by the percentage of positive pixel intensity values in their control samples. The result was shown as a fold change to compare the difference in VSMC coverage, vascular density, and nerve density. The degree of αSMA^+^ VSMC coverage as a percentage of the total length of small-to-medium in the peripheral region and medium-to-large diameter remodeled blood vessels in the central region was analyzed (five medium-to-large diameter and five small-to-medium blood vessels per ear skin). CD11b^+^ inflammatory cells were manually counted from at least three magnified images each from the peripheral and central region per ear skin.

For hematoxylin-eosin staining, ears were dissected and fixed in 10% formalin at room temperature for at least one overnight.

Gross pictures of the ear skin dissection (Fig. 1B and Supplemental Fig. 1B) were captured by stereomicroscope Zeiss SteREO Discovery.V12 (Zeiss).

All experiments were performed in accordance with the NIH laboratory safety.

### Statistical Analyses

Number of mice is indicated as “n” in Figure Legends. Data are presented as means ± S.E.M. Statistical significance between two groups was assessed using a two-tailed Student’s *t* test. P < 0.05 was considered to be significant. The investigators were not blinded during confocal imaging but quantifications in ImageJ were performed blind.

## Electronic supplementary material

Supplementary Information
