# Supplementary material for: Whole-Mount Adult Ear Skin Imaging Reveals Defective Neuro-Vascular Branching Morphogenesis in Obese and Type 2 Diabetic Mouse Models
Source: Sci Rep. 2018 Jan 11;8:430. doi: 10.1038/s41598-017-18581-7 (PMC5764985; doi:10.1038/s41598-017-18581-7)
Supplement: Supplementary file 1 — Supplementary Information [file 41598_2017_18581_MOESM1_ESM.pdf]

SUPPLEMENTARY INFORMATION

WHOLE-MOUNT ADULT EAR SKIN IMAGING REVEALS DEFECTIVE NEURO-VASCULAR BRANCHING MORPHOGENESIS IN OBESE AND TYPE 2 DIABETIC MOUSE MODELS

Tomoko Yamazaki, Wenling Li, Ling Yang, Ping Li, Haiming Cao, Sei-ichiro Motegi, Mark C. Udey, Elise Bernhard, Takahisa Nakamura, and Yoh-suke Mukouyama

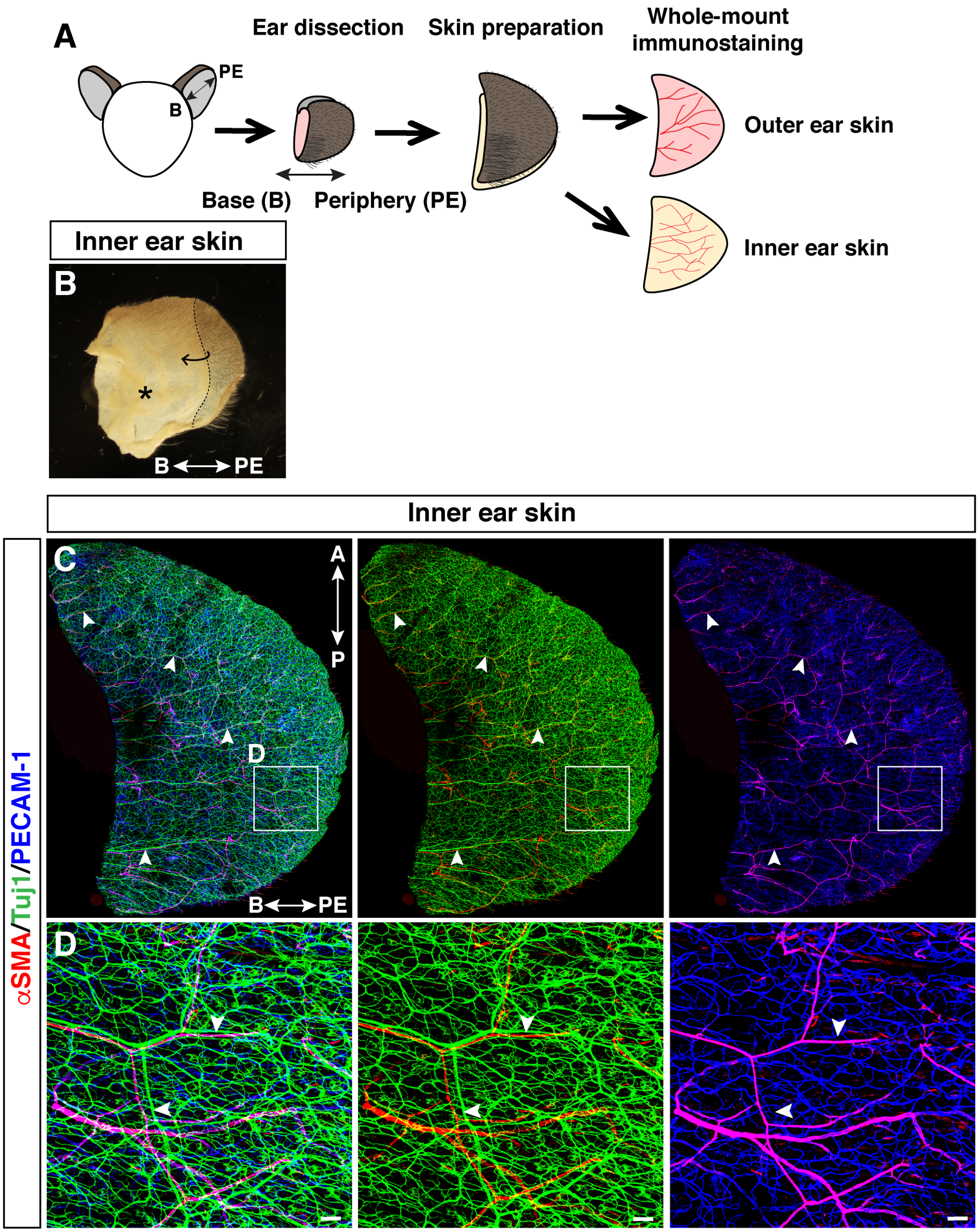
A
Ear dissection
Skin preparation
Whole-mount immunostaining
PE
B
Base (B)
Periphery (PE)
Outer ear skin
Inner ear skin
Inner ear skin
B
B ⟷ PE
Inner ear skin
C
A
P
D
B ⟷ PE
D
αSMA/Tuj1/PECAM-1

Supplementary Figure 1. Whole-mount immunofluorescence confocal microscopy of adult inner ear skin

(A) Diagram of dissection of outer and inner parts of ear skin. (B) First, outer part of the ear skin was peeled away from the cartilage (not shown). Next, the cartilage (asterisk) was peeled off from inner skin before staining. (C) Inner ear skin was stained with antibodies to a VSMC marker αSMA (red), a pan-neuronal marker Tuj1 (green), and a pan-endothelial marker PECAM-1 (blue). The boxed regions of (C), peripheral regions of the inner ear skin, are magnified in (D). Arrowheads indicate the alignment of small-diameter nerves and VSMC-covered remodeled blood vessels. The orientation of base (B)-periphery (PE) axis and anterior (A)-posterior (P) axis is shown. Scale bars, 100 μm.
